# Impact of penalizing factor in a block-sequential regularized expectation maximization reconstruction algorithm for ^18^F-fluorocholine PET-CT regarding image quality and interpretation

**DOI:** 10.1186/s40658-019-0242-2

**Published:** 2019-03-21

**Authors:** Mimmi Bjöersdorff, Jenny Oddstig, Nina Karindotter-Borgendahl, Helén Almquist, Sophia Zackrisson, David Minarik, Elin Trägårdh

**Affiliations:** 1 Clinical Physiology and Nuclear Medicine, Skåne University Hospital and Lund University, Malmö, Sweden; 2 Radiation Physics, Skåne University Hospital and Lund University, Carl Bertil Laurells gata 9, SE-205 02 Malmö, Sweden; 3 Medical Radiology, Skåne University Hospital and Lund University, Carl Bertil Laurells gata 9, SE-205 02 Malmö, Sweden; 4 Wallenberg Center for Molecular Medicine, Lund University, Lund, Sweden

**Keywords:** Image quality, Oncology, ^18^F-FCH, Protocol optimization, Q.Clear

## Abstract

**Background:**

Recently, the block-sequential regularized expectation maximization (BSREM) reconstruction algorithm was commercially introduced (Q.Clear, GE Healthcare, Milwaukee, WI, USA). However, the combination of noise-penalizing factor (β), acquisition time, and administered activity for optimal image quality has not been established for ^18^F-fluorocholine (FCH). The aim was to compare image quality and diagnostic performance of different reconstruction protocols for patients with prostate cancer being examined with ^18^F-FCH on a silicon photomultiplier-based PET-CT. Thirteen patients were included, injected with 4 MBq/kg, and images were acquired after 1 h. Images were reconstructed with frame durations of 1.0, 1.5, and 2.0 min using β of 150, 200, 300, 400, 500, and 550. An ordered subset expectation maximization (OSEM) reconstruction with a frame duration of 2.0 min was used for comparison. Images were quantitatively analyzed regarding standardized uptake values (SUV) in metastatic lymph nodes, local background, and muscle to obtain contrast-to-noise ratios (CNR) as well as the noise level in muscle. Images were analyzed regarding image quality and number of metastatic lymph nodes by two nuclear medicine physicians.

**Results:**

The highest median CNR was found for BSREM with a β of 300 and a frame duration of 2.0 min. The OSEM reconstruction had the lowest median CNR. Both the noise level and lesion SUV_max_ decreased with increasing β. For a frame duration of 1.5 min, the median quality score was highest for β 400-500, and for a frame duration of 2.0 min the score was highest for β 300-500. There was no statistically significant difference in the number of suspected lymph node metastases between the different image series for one of the physicians, and for the other physician the number of lymph nodes differed only for one combination of image series.

**Conclusions:**

To achieve acceptable image quality at 4 MBq/kg ^18^F-FCH, we propose using a β of 400-550 with a frame duration of 1.5 min. The lower β should be used if a high CNR is desired and the higher if a low noise level is important.

**Electronic supplementary material:**

The online version of this article (10.1186/s40658-019-0242-2) contains supplementary material, which is available to authorized users.

## Background

Positron emission tomography with computed tomography (PET-CT) is a powerful and widely spread medical imagining technique primarily used in oncology [1, 2]. Previous PET-CT scanners were built with scintillation crystals coupled to photomultiplier tubes. Recently, a novel generation of PET-scans, with a silicon photomultiplier-based technology, was introduced, which has the potential to increase detection of pathology, primarily through higher sensitivity [3–5]. Concurrently, improved reconstruction methods have been described and one such method is the block-sequential regularization expectation maximization algorithm (BSREM) [6], with the commercial name Q.Clear (GE Healthcare, Milwaukee, WI, USA) [4, 7]. This method was developed to improve the quantitative accuracy [8]. When using conventional iterative reconstruction algorithms such as ordered subset expectation maximization (OSEM), the accuracy of the measured standardized uptake values (SUV) of lesions improves when the number of iterations is increased. However, this also increases noise and can limit the detection of small lesions. Stopping the iterative process after a limited number of iterations, in order to reduce noise, leads to an underestimation of SUV in smaller lesions [7]. The BSREM algorithm allows full convergence while suppressing noise, via a penalty term. This increases the SUV, particularly in small lesions versus conventional reconstruction methods [8], while still maintaining relatively low noise levels. The algorithm is based on the following objective function:

1 $$ \Phi \left(\boldsymbol{x}\right)={\sum}_i{y}_i\bullet \mathit{\log}\left({\left[P\boldsymbol{x}\right]}_i+{b}_i\right)-\left({\left[P\boldsymbol{x}\right]}_i+{b}_i\right)-\beta \bullet R(x) $$

where *y*_i_ is the measured data, *P* is the system matrix containing the detection probabilities, *b* indicates the estimated background events of randoms and scatter, and *x* is the image estimate. β indicates the global smoothing parameter controlling the overall impact of the relative difference penalty term *R*(*x*). The BSREM algorithm is used to maximize the objective function Φ.

The influence of the β factor has been investigated for ^18^F-fluorodeoxyglucose [9–11] and 18F-fluciclovinell [12], but not for ^18^F-fluorocholine (FCH). ^18^F-FCH has been used for the last 20 years to stage patients with high-risk prostate cancer [13]. The new hardware and software technologies in PET-CT can potentially increase the accuracy and early detection of small lesions such as lymph node metastasis, which is vital in the management of prostate cancer [14]. Due to the different uptake mechanisms and pathological conditions, it cannot be expected that the same reconstruction parameters as for ^18^F-fluorodeoxyglucose can be used for ^18^F-FCH. Also, to our knowledge, no study with blinded interpretation of images with different reconstruction parameters for BSREM has been done before.

The aim of this study was to evaluate ^18^F-FCH images from a novel silicon photomultiplier-based PET-CT (Discovery MI, GE Healthcare, Milwaukee, WI, USA) by assessing the image quality and diagnostic performance of BSREM for different β values as well as for different frame durations per bed position.

## Methods

### PET-CT-system

A Discovery MI PET-CT installed in 2017 was used to carry out the examinations. The system uses lutetium-yttrium oxyorthosilicate crystals (crystal size 4.0 × 5.3 × 25 mm^3^) coupled to an array of SiPM. The PET-detector has an axial field of view of 20 cm and an overlap of 24%. The sensitivity according to NEMA standards is 13 cps/kBq. The system has a 128-slice CT.

### Clinical data

We enrolled 13 patients with biopsy-verified high-risk prostate cancer who were referred for an ^18^F-FCH PET-CT at Skåne University Hospital, Sweden. The study is regarded as development, and all images were anonymized prior to the analysis. Therefore, no ethical board evaluation was required according to Swedish law. The study complies with the Declaration of Helsinki.

### Imaging data

^18^F-FCH (4 MBq/kg) was administrated to the patients via a single intravenous injection after a minimum of 4 h fasting. The images were acquired after an accumulation time of 60 min. Images were acquired from the upper thigh to the base of the skull with a frame duration of 2.0 min per bed position. The PET data was obtained and stored in list mode. The images were reconstructed using BSREM including time-of-flight and point spread function with a 256 × 256 matrix (pixel size 2.7 × 2.7 mm^2^, slice thickness 2.8 mm). Different β values (150, 200, 300, 400, 500, and 550) and different frame durations (1.0, 1.5, and 2.0 min) were used. For comparison, an OSEM reconstruction including time-of-flight and point spread function, 4 iterations, 16 subsets, standard *z*-axis filter and a 6 mm Gaussian post filter, a 256 × 256 matrix with a frame duration of 2.0 min was used. Thus, 19 different image series were obtained from each patient.

A diagnostic CT was performed with tube current modulation applied, adjusting the tube current for each patient, with a noise index of 42.25. A tube voltage of 100 kV was used for body mass index (BMI) ≤ 30, and 120 kV was used for BMI > 30. An adaptive statistical iterative reconstruction technique was used.

### Quantitative image analysis

A region of interest (ROI) was drawn over one pathologic pelvic lymph node per patient. The size of the selected lymph nodes was measured in a trans-axial CT slice (short- and long-axis). ROIs were also defined in local background adjacent to the lymph node and in muscle (rectus femoris or vastus lateralis muscles) for noise estimation. The Advantage Workstation version 7 (GE Healthcare, Milwaukee, WI, USA) was used to define the ROIs. The same ROIs were used for all 19 image series. The mean, maximum, and standard deviation (SD) SUV in the ROIs were calculated. The contrast-to-noise ratio (CNR) were calculated as follow:

2 $$ \mathrm{CNR}=\frac{{\mathrm{SUV}}_{\max, \mathrm{lesion}}-{\mathrm{SUV}}_{\mathrm{mean}\ \mathrm{in}\ \mathrm{local}\ \mathrm{background}}}{{\mathrm{SUV}}_{\mathrm{SD}\ \mathrm{muscle}\ \mathrm{background}}} $$

The local background SUV_mean_ was calculated by using a ROI that comprised of the set difference of a local background ROI that was slightly larger and covering the lymph node ROI and the lymph node ROI itself. The noise level was defined as coefficient of variation (COV) and calculated as Eq. 3.

3 $$ \mathrm{COV}=\frac{{\mathrm{SUV}}_{\mathrm{SD}\ \mathrm{muscle}}}{{\mathrm{SUV}}_{\mathrm{mean}\ \mathrm{muscle}}} $$

### Qualitative image analysis

#### Pilot study

Three patients were randomly selected for visual assessment of image quality. The examinations were graded on a scale from 1 to 5 (1 = unacceptable image quality, 2 = less than acceptable image quality, 3 = acceptable image quality, 4 = high image quality, and 5 = very high image quality). This evaluation was performed by one experienced nuclear medicine physician. The six reconstructions with highest median scores plus the image based on OSEM (for comparison) were chosen for further qualitative evaluation described below. This procedure was performed to reduce the number of images evaluated and interpreted.

#### Assessment of image quality

The seven best image series from the pilot study were evaluated for image quality (scale 1–5, described above). The remaining ten patients were evaluated in a blinded fashion by five nuclear medicine physicians. The physicians were not aware of the reconstruction parameters or which of the ten patients they evaluated, but were provided information regarding the indication for the examination (staging of high-risk prostate cancer).

#### Interpretation of images

Two experienced nuclear medicine physician independently interpreted the image series in a random manner as above, by assessing the number of suspected metastases in pelvic lymph nodes.

### Statistical analysis

Median CNR, noise level, and SUV_max_ were calculated for a single reconstruction setting over all patients. For ranking of CNR, the CNR values for all 19 image series were ranked (1–19; the highest to lowest CNR) for each patient and then the mean rank for all patients was calculated. The Friedman ranking test was used to test for differences in CNR, noise level, and lesion SUV_max_ as well as the number of metastatic lymph nodes for the different image series. A Wilcoxon signed-rank test was used as the post-hoc test when a statistical significance was found. Differences in image quality for different reconstruction parameters were tested using Kruskal-Wallis test, and post-hoc analysis performed using Mann Whitney *U* test. Bonferroni corrections for multiple tests were used, and the adjusted *p* values are shown throughout the manuscript. Statistical significance was considered for *p* less than 0.05. Statistical analyses were performed using IBM SPSS version 25 (IBM, Armonk, NY, USA).

## Results

### Patients

Thirteen patients were enrolled and examined with whole-body ^18^F-FCH PET-CT. The mean weight was 86.2 ± 13.6 kg (range 70–120 kg); the mean BMI was 27.1 ± 3.6 (range 23.1–34.3). The mean administrated ^18^F-FCH was 4.0 ± 1.2 MBq/kg (range 3.7–4.3 MBq/kg), and the mean accumulation time was 63 ± 4 min (range 59–70 min).

### Quantitative analysis

The lymph nodes selected for the quantitative analysis had a median size of 6 × 9 mm (short- and long-axis, respectively) with a range of 4–21 mm (short-axis) × 6–21 mm (long-axis). Only two lymph nodes exceeded a short axis measurement of 10 mm. The two largest lymph nodes had a center of lower attenuation on CT, but still had a homogeneous ^18^F-FCH uptake. The other lymph nodes had a homogenous appearance on CT.

**Fig. 1:**
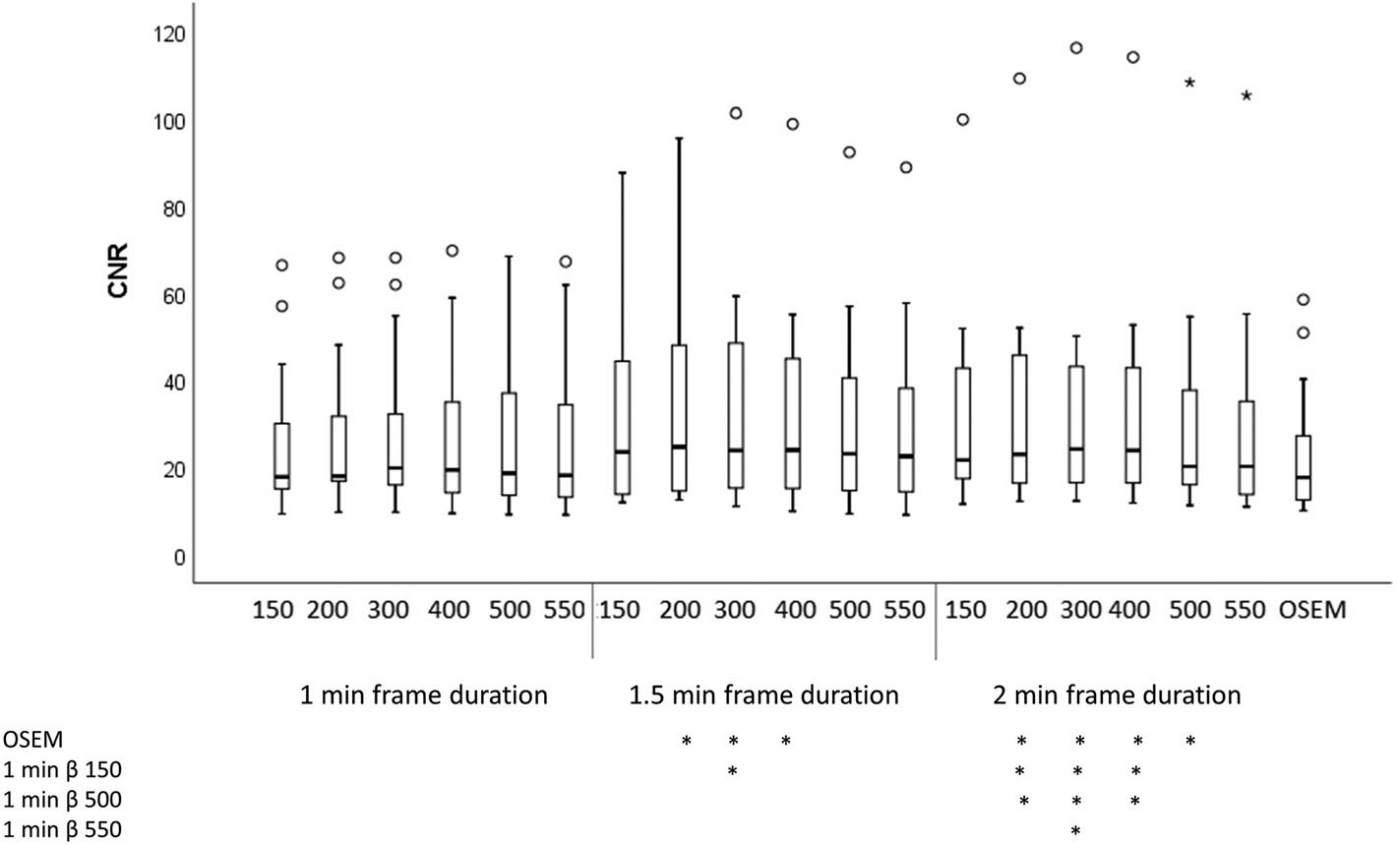
Boxplot showing data for CNR in pathologic lymph nodes. The median is visible through the thick line in the middle of the boxes. The first and third quartiles are shown through the top and bottom box lines. The whiskers extend to 1.5 times the height of the box, or if no case has a value in that range, to the minimum and maximum values. The circles in the graph are outliers, values outside the definition of the whiskers. The stars in the graph are extreme outliers, values of more than three times the height of the box. The stars below the graph indicate the combinations of reconstruction parameters that reached statistical significance

**Fig. 2:**
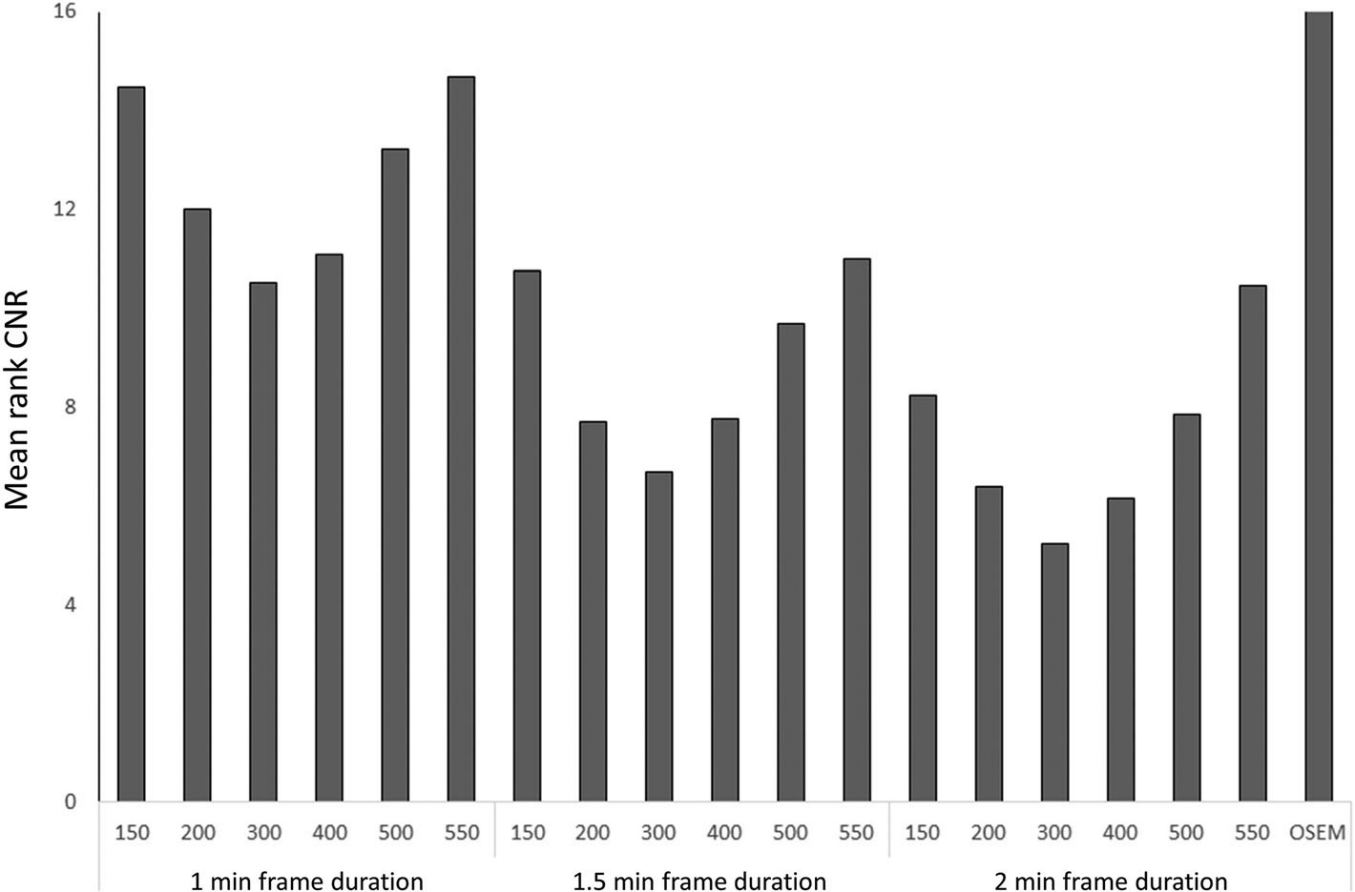
Mean rank of CNR in pathologic lymph nodes. Ranks (1–19 where 1 is the best) were calculated for all patients and then averaged

The highest median CNR was found with BSREM and a frame duration of 2.0 min with β 300 (Fig. 1). The OSEM reconstruction had the lowest median CNR. The ranking (Fig. 2) shows that the best CNR for all frame durations was when a β of 300 was used. The reconstruction with a frame duration of 2.0 min and β 300 had a significantly higher CNR than OSEM (*p* > 0.0001). It had also a significantly higher CNR than image series with β 150, 500, and 550 with a frame duration of 1.0 min (*p* = 0.05, *p* = 0.05, and *p* = 0.003 respectively). All other CNR comparisons between image series were not statistically significant. The *p* values for all different series combinations and CNR can be found in Additional file 1: Table S1.

**Fig. 3:**
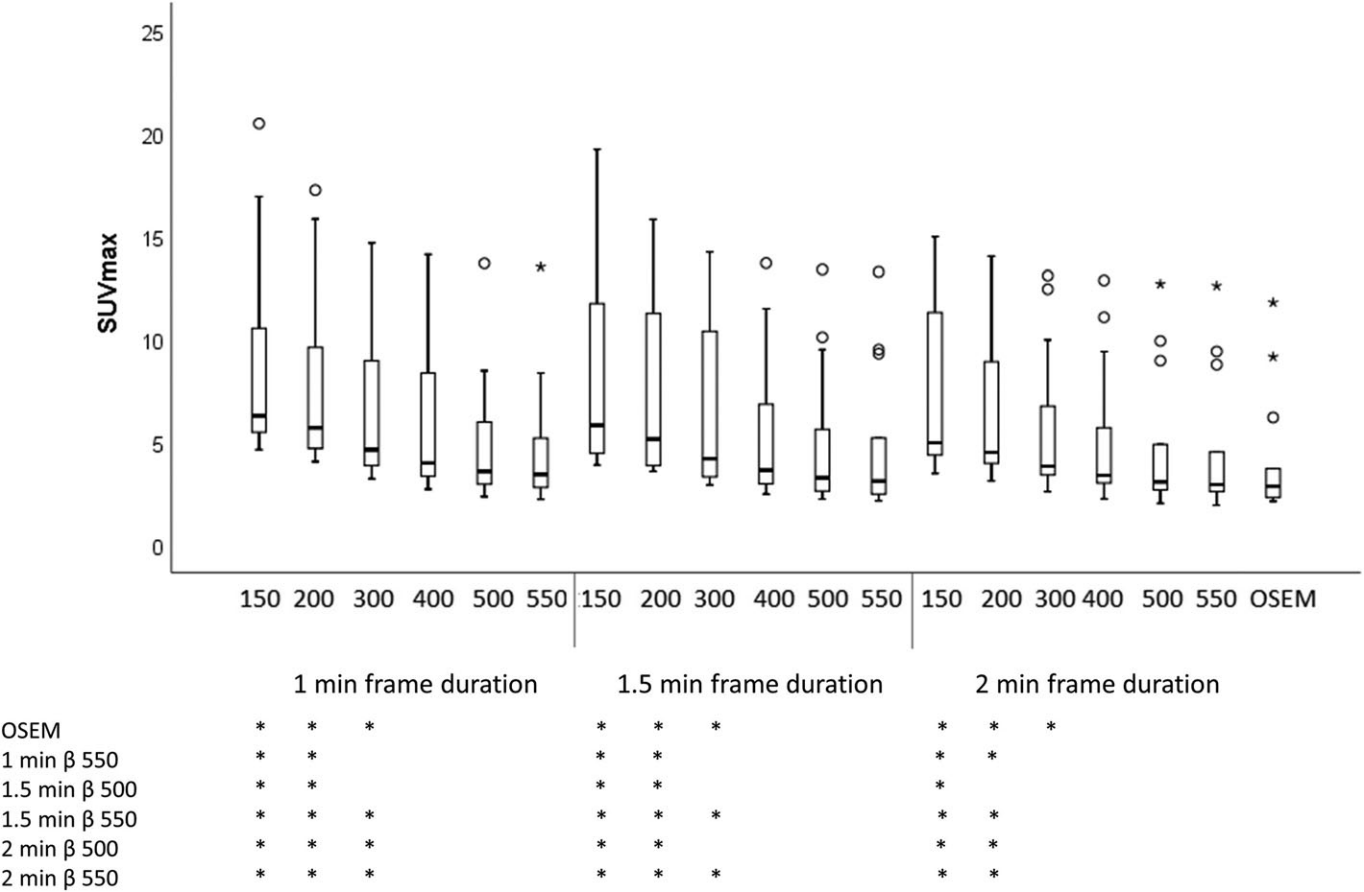
Boxplot for SUV_max_ in pathologic lymph nodes. Stars below the graph indicate the combinations of reconstruction parameters that reached statistical significance

The highest median SUV_max_ was found with a β of 150 and 1.0 min. The lowest median SUV_max_ was found with OSEM (Fig. 3). The SUV_max_ was significantly higher for β 150-200 (all frame durations) compared to OSEM, β 550 with a frame duration of 1.0 min, and β of 500-550 with frame durations of 1.5–2.0 min. The *p* values for all different series combinations and SUV_max_ are found in Additional file 1: Table S1.

**Fig. 4:**
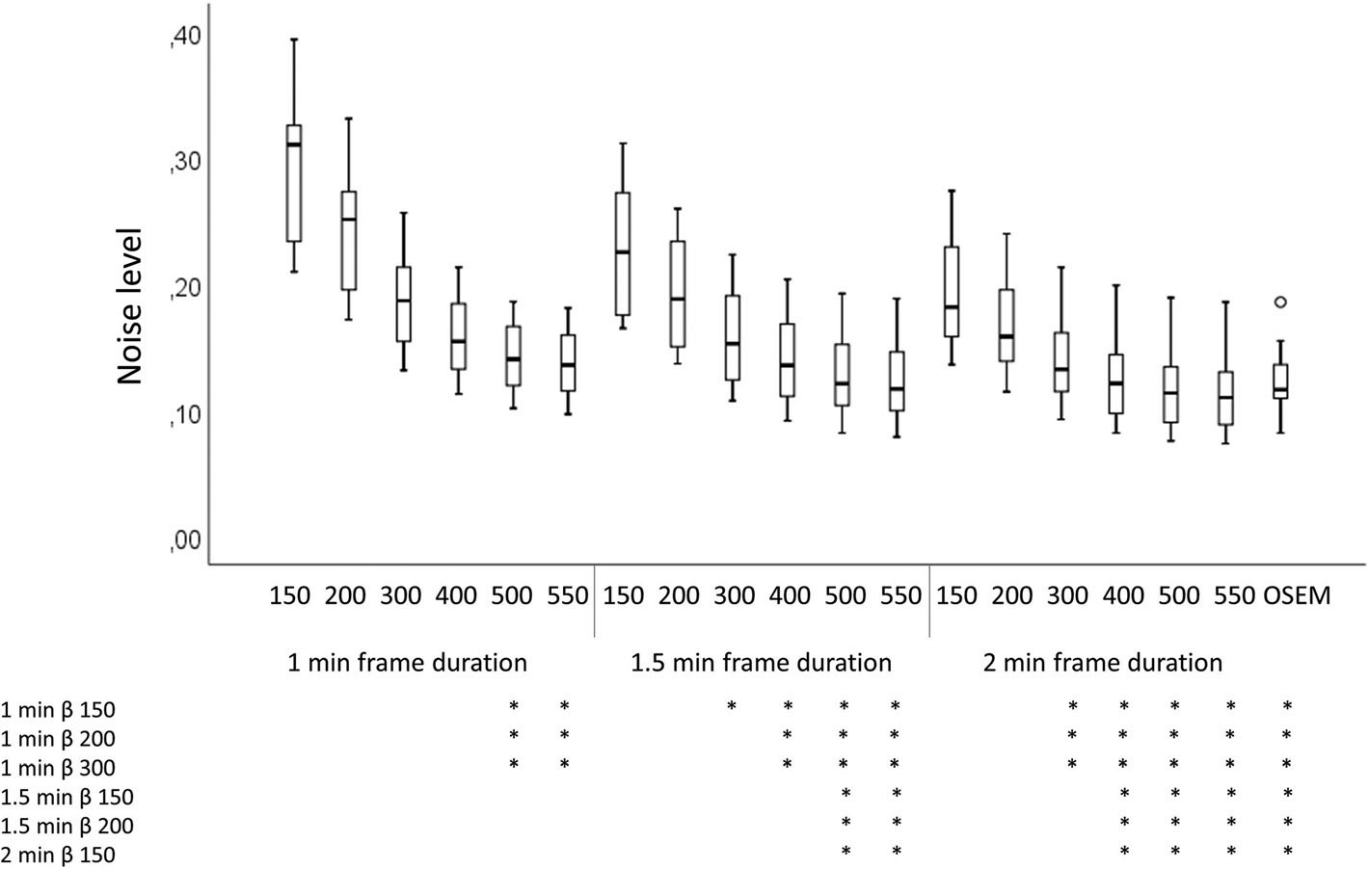
Boxplot for the noise level in muscle. Stars below the graph indicate the combinations of reconstruction parameters that reached statistical significance

The lowest noise level was found for a β of 550 with frame duration of 2.0 min. The highest was with a β of 150 and frame duration of 1.0 min (Fig. 4). The OSEM reconstruction had a similar noise level as β 550 and frame duration of 1.5 min and β 500 with frame duration of 2.0 min. The *p* values for all different series combinations and noise level are found in Additional file 1: Table S1.

**Fig. 5:**
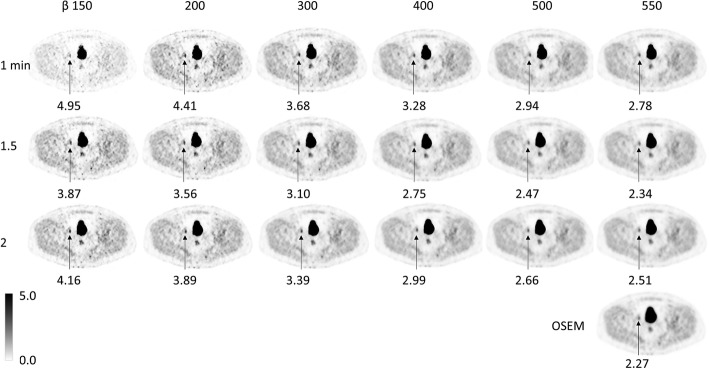
Transversal images in the pelvis from one of the patients for the different reconstruction series. Arrow indicate the pathologic lymph node and the lesion SUV_max_

Figure 5 shows a representative example of image quality and different lesion SUV_max_ values for all series.

### Qualitative analysis

#### Pilot study

The best scores of image quality for the first three patients were the combination of frame durations 1.5 and 2.0 min and β values of 300, 400, 500, and 550 as well as OSEM (Table 1). BSREM with β 500 and 550 had identical ranking and similar visual appearance; therefore, we proceeded with only β 300, 400, and 500 and OSEM in order to reduce the number of images to evaluate. Thus, these seven series were used for further qualitative evaluation.

**Table 1:**
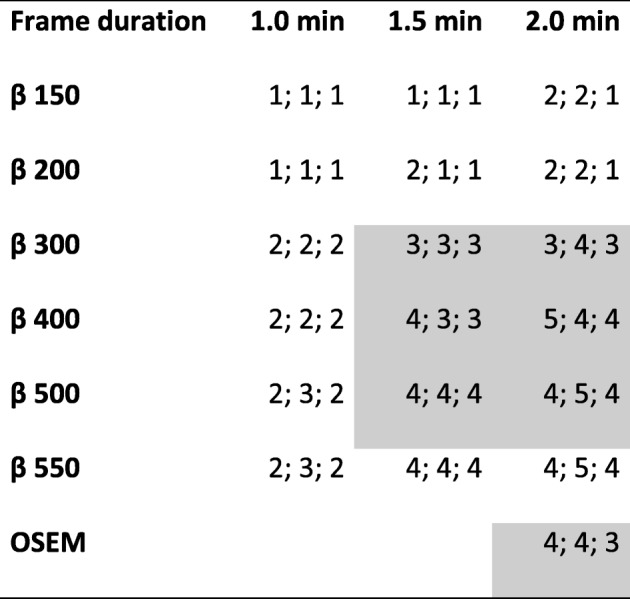
Image quality in pilot study

Image quality in pilot study. Image quality in 18F-FCH PET-CT examinations (graded 1–5 where 1 = unacceptable image quality and 5 = very high image quality) for the three patients in the pilot study for all combinations of β and frame duration as well OSEM. The combinations shown in grey were further evaluated regarding image quality and lymph node metastases

#### Assessment of image quality

The median quality score was highest (3/5 = acceptable image quality) for β 400-500 with a frame duration of 1.5 min and for β 300-500 with a frame duration of 2.0 min (Table 2). The reconstruction with β 300 and a frame duration of 1.5 min had significantly lower image quality compared to all other image series except OSEM (*p* = 0.007 for comparison with β 400 with 1.5 min, *p* < 0.001 for β 500 with 1.5 min, *p* < 0.001 for β 300-500 with 2.0 min). Also, the OSEM reconstruction had significantly lower image quality compared to β 500 with 1.5 min and (*p* = 0.005), and β 500 with 2.0 min (*p* = 0.004), and β 500 with 2.0 min (*p* = 0.003). No other combinations were found statistically significant.

**Table 2:** Assessment of image quality

	Observers (median, range)					
	#1	#2	#3	#4	#5	Overall
1.5 min, β 300	2 (1–3)	2 (2–4)	2 (1–3)	3 (2–4)	2 (1–2)	2
1.5 min, β 400	3 (1–3)	3 (2–4)	2.5 (2–5)	3 (2–4)	2 (2–3)	3
1.5 min, β 500	3 (3–4)	3 (3–4)	3 (3–4)	3 (2–4)	3 (2–4)	3
2.0 min, β 300	2 (1–3)	3.5 (2–4)	2 (1–5)	4 (3–4)	2 (2–3)	3
2.0 min, β 400	3.5 (1–4)	3 (3–4)	3 (2–4)	3 (2–4)	3 (2–4)	3
2.0 min, β 500	3 (3–4)	3 (2–4)	3 (3–4)	2.5 (2–3)	3.5 (2–4)	3
2.0 min, OSEM	3.5 (2–4)	3 (2–3)	2.5 (1–3)	2 (2)	2.5 (2–3)	2.5

Assessment of image quality. Assessment of median (and range) image quality in ^18^F-FCH PET-CT examinations by five observers by grading images on a scale 1–5 (where 1 = unacceptable image quality and 5 = very high image quality) for the remaining ten patients for each image. The overall median image quality score for each series of reconstructions is also presented

#### Interpretation of images

**Fig. 6:**
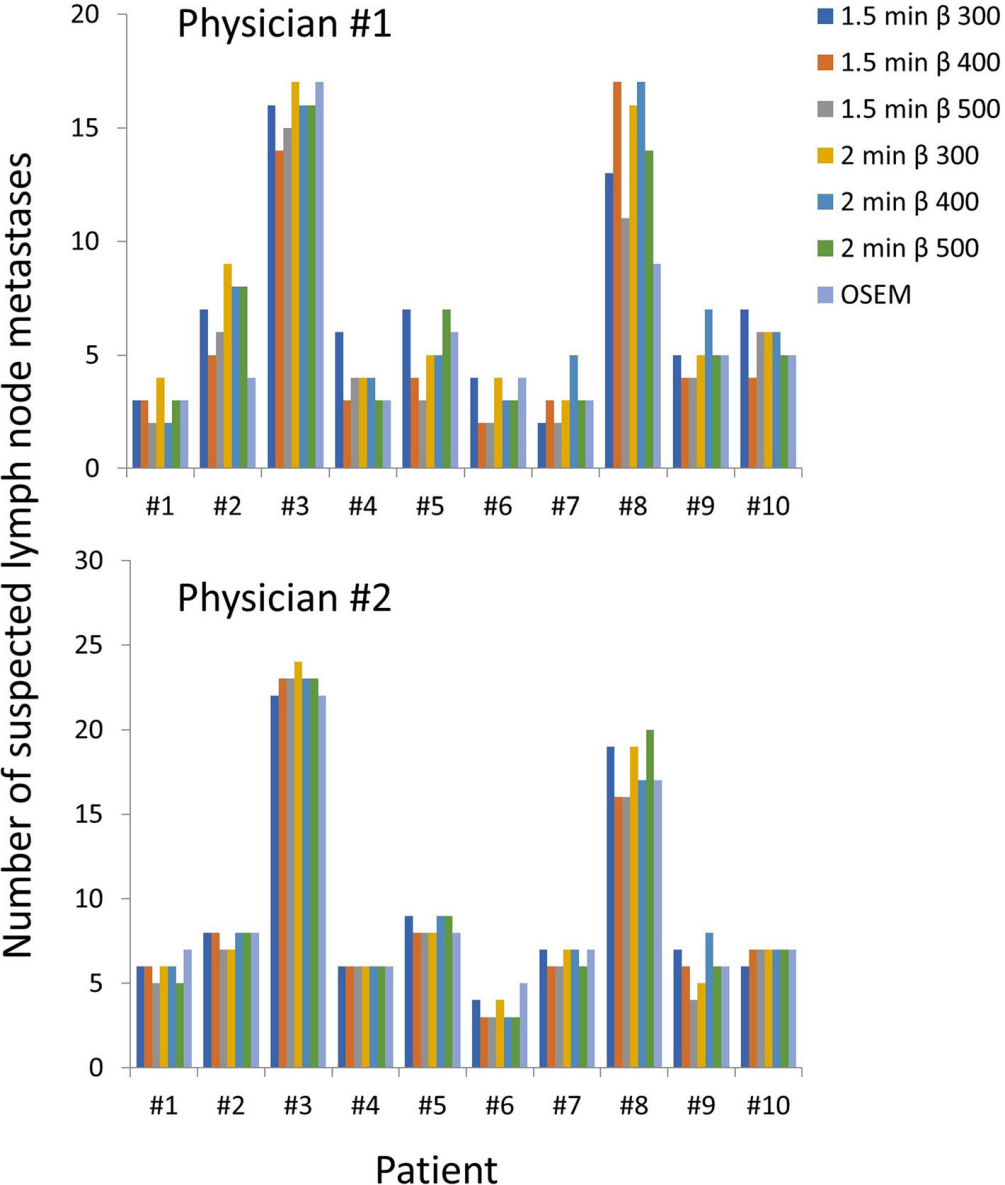
Number of suspected metastatic lymph nodes for different image series. The graphs show the number of lymph nodes detected for the two nuclear medicine physicians (upper and lower graph) for the ten patients. The only significant difference was between 1.5 min with β 500 and 2.0 min with β 300 for physician #1

Figure 6 shows the number of suspected lymph node metastases found for the different image series and patients. For one of the physicians, there was a statistically significant difference of lymph nodes detected between β 500 with 1.5 min and β 300 with 2.0 min (*p* = 0.040). No other combinations were statistically significant. For the other physician, there were no statistically significant differences in the number of suspected lymph nodes between the different series (*p* = 0.106).

## Discussion

The highest CNR was found for β 300 with a frame duration of 2.0 min and the lowest was found for the OSEM reconstruction. The SUV_max_ and noise level decreased with increasing β. For a frame duration of 1.5 min, the highest subjective image quality was found using a β of 400-500. For a frame duration of 2 min, the best quality was found with β 300-500. There were no significant differences in the number of suspected lymph node metastases, except for one combination of image series for one of the physicians, which is probably a random finding.

Regarding the ranking of CNR, there was a “U”-shape for each frame duration in relation to the β factor (Fig. 2). To obtain a high CNR, it is important to have both a relatively high lesion SUV_max_ and a relatively low noise level. The low CNR for low β values is due to a high noise level, whereas the low CNR for high β values is due to a low lesion SUV_max_. The highest median value and best ranking for CNR was found with β 300 and frame duration of 2.0 min_._ The CNR increased with increasing frame duration due to lower noise levels in these images.

SUV_max_ decreases with increasing β, which corresponds to previous studies [9, 11, 12]. Noisy images (generally images series with a frame duration of 1.0 min) have a SUV_max_ that is very noise-dependent. Here, the SUV_max_ was lower for a frame duration of 2.0 min compared to image series with frame durations of 1.0 min and 1.5 min, for the same β value, and this is due to the higher noise levels in the latter. The use of SUV_peak_ is generally considered less noise dependent but since most of the lesions in this study were smaller than 1 cm^3^, SUV_peak_ was not a relevant measure. However, SUV_max_ in sub-centimeter lesions when using point-spread function reconstructed PET images has been shown to not be reliable due to artifacts [15].

The image series reconstructed with OSEM was used for comparison since it is a well-established reconstruction method. The number of iterations, subsets, and post-filter in the OSEM reconstruction used in this study originate from an initial optimization when the PET-CT system was installed and was not further optimized since that was not the purpose with the study. The OSEM used in this study is designed in accordance with the updated EARL accreditation specifications [16]. If different reconstruction parameters for OSEM are used, different values for CNR, SUV_max_, and noise are expected.

The pilot study was conducted to obtain relevant reconstruction parameters and to identify a feasible number of image series for the subsequent qualitative analysis. Analysis of all series combinations would be desirable but was not feasible. The pilot study showed that the median image quality of whole-body ^18^F-FCH was less than acceptable for frame duration of 1 min, regardless of β, as well as for 1.5 and 2.0 min with β of 150-200. The definition of good image quality differed between the five observers, due to differences in the preferred or accepted image contrast and noise level. For example, as seen in Table 2, physician #4 prefers noisier high contrast images compared to the other physicians. The image quality assessment showed a median quality of 3 (= acceptable image quality) for all image series except OSEM and for β 300 with 1.5 min/frame. The relatively low variation in the combinations of frame duration and β values may have reduced the range of scores for image quality. In order to obtain a median score of high or very high image quality, we expect that the acquisition time needs to be substantially longer.

There was no significant difference in the number of suspected metastatic lymph nodes with different reconstructions or different frame durations, except for one combination of image series for one of the physicians, which is probably a random finding. Although only a small number of patients were analyzed, this indicates that all tested image series provide sufficient image quality to interpret the images.

Previous studies on BSREM with ^18^F-fluorodeoxyglucose [9–11, 17, 18] and ^18^F-fluciclovine [12] exist. However, to the best of our knowledge, such a study has never been done before for ^18^F-FCH. In patients with prostate cancer, it is important to be able to detect relatively low ^18^F-FCH uptake in small lymph nodes, which is why optimization of reconstruction parameters for this tracer is important and may not be the same as for other tracers and cancer types. Also, to our knowledge, this is the only study performing blinded interpretations of images with different reconstruction parameters for BSREM. This makes it possible to recommend a range of acceptable β, where the clinical image interpretation is not affected.

In this study, the patients were administered with 4 MBq/kg and scanned with a frame duration of 1.0, 1.5, and 2.0 min. The activity and frame duration are interchangeable: 8 MBq/kg with a frame duration of 1.0 min is to a close approximation the same as 4 MBq/kg and 2.0 min/frame. Consider the activity-time (AT), defined as the product of the administered activity per unit body weight and the frame duration (MBq/kg*min), assuming a one hour delay between administration and scan time, then the findings suggest that the image quality is substandard for AT 4 MBq/kg*min regardless of β value. It is also not sufficient for AT 6 MBq/kg*min with β of 150-300 and AT 8 MBq/kg*min with β of 150-200. Thus, any of these combinations should not be used. The remaining combinations of AT and β values (AT 6 MBq/kg*min with β of 400-550, AT MBq/kg*min 8 with β of 300-550) have reasonably good CNR, noise level, and subjective image quality. The number of suspected lymph node metastases identified does not differ; thus, any of these combinations can be used. However, it seems sufficient to use an AT of 6 MBq/kg*min, which is preferable.

## Limitations

The findings should be viewed in light of some limitations. First, only few patients were analyzed. The goal here was to test many reconstructions—more patients would result in a very large number of images to assess, which was not possible. Second, due to the large number of images, only two nuclear medicine physicians were available to interpret the images. We did not evaluate the inter-observer variability, since this was not the aim of the study. Inter-observer variability for FCH PET-CT has previously been shown to be moderate for local recurrence in the prostate (Fleiss’ kappa 0.55) and to be good for lymph node metastases (Fleiss’ kappa 0.89) [19]. Third, in order to minimize the risk of the physicians recognizing the patients when interpreting the images, they performed the image interpretation over several weeks. However, the risk is not completely eliminated. Forth, only three patients were evaluated in the pilot study, and it is possible that the images from these patients are not representative for all ^18^F-FCH examinations. Fifth, depending on the reconstruction parameters used for OSEM, different results can be obtained. Therefore, the results of this study cannot be used for comparison between BSREM and OSEM in general.

## Conclusion

To achieve high image quality at 4 MBq/kg ^18^F-FCH, we propose using a β of 400-550 with a frame duration of 1.5 min. The lower β can be used if a high CNR is desired and the higher β if a low noise level is more important for the physician interpreting the images. For these reconstruction combinations, there was no statistically significant difference in the number of suspected lymph node metastases found.

## Additional file

Additional file 1: **Table S1.** *P* values from the post-hoc test for the different combinations of image series for CNR, SUV_max_ and noise. Those combinations not shown in the table all had *p* = 1.000. All shown *p*-values were adjusted with the Bonferroni method due to multiple comparisons. *P* values marked in gray are statistically significant. (DOCX 19 kb)

## Abbreviations

AT: Activity-time
BMI: Body mass index
BSREM: Block-sequential regularized expectation maximization algorithm
CNR: Contrast-to-noise ratio
COV: Coefficient of variation
CT: Computed tomography
FCH: Fluorocholine
OSEM: Ordered subset expectation maximization
PET-CT: Positron emission tomography with computed tomography
ROI: Region of interest
SD: Standard deviation
SUV: Standard uptake values
β: Beta value
