# Supplementary material for: Impact of penalizing factor in a block-sequential regularized expectation maximization reconstruction algorithm for 18F-fluorocholine PET-CT regarding image quality and interpretation
Source: EJNMMI Phys. 2019 Mar 21;6:5. doi: 10.1186/s40658-019-0242-2 (PMC6428870; doi:10.1186/s40658-019-0242-2)
Supplement: Supplementary file 1 — Table S1. P values from the post-hoc test for the different combinations of image series for CNR, SUVmax and noise. Those combinations not shown in the table all had p = 1.000. All shown p-values were adjusted with the Bonferroni method due to multiple comparisons. P values marked in gray are statistically significant. (DOCX 19 kb) [file 40658_2019_242_MOESM1_ESM.docx]

**Table S1.** P-values from the post-hoc test for the different combinations of image series for CNR, SUV_max_ and noise. Those combinations not shown in the table all had p=1.000. All shown p-values were adjusted with the Bonferroni method due to multiple comparisons. P-values marked in grey are statistically significant.

| **Sample 1** | **Sample 2** | **CNR** | **SUV_max_** | **Noise (COV)** |
| --- | --- | --- | --- | --- |
| OSEM | 1 min β 150 | 1.000 | <0.001 | <0.001 |
| OSEM | 1 min β 200 | 1.000 | <0.001 | <0.001 |
| OSEM | 1 min β 300 | 1.000 | 0.001 | <0.001 |
| OSEM | 1 min β 400 | 1.000 | 0.149 | 1.000 |
| OSEM | 1.5 min β 150 | 1.000 | <0.001 | <0.001 |
| OSEM | 1.5 min β 200 | 0.025 | <0.001 | <0.001 |
| OSEM | 1.5 min β 300 | 0.004 | 0.001 | 1.000 |
| OSEM | 1.5 min β 400 | 0.029 | 0.275 | 1.000 |
| OSEM | 1.5 min β 500 | 0.653 | 1.000 | 1.000 |
| OSEM | 2 min β 150 | 0.065 | <0.001 | 0.001 |
| OSEM | 2 min β 200 | 0.002 | <0.001 | 1.000 |
| OSEM | 2 min β 300 | <0.001 | 0.031 | 1.000 |
| OSEM | 2 min β 400 | 0.001 | 1.000 | 1.000 |
| OSEM | 2 min β 500 | 0.033 | 1.000 | 1.000 |
| 1 min β 150 | 1 min β 400 | 1.000 | 1.000 | 0.204 |
| 1 min β 150 | 1 min β 500 | 1.000 | 1.000 | 0.002 |
| 1 min β 150 | 1 min β 550 | 1.000 | <0.001 | <0.001 |
| 1 min β 150 | 1.5 min β 200 | 0.370 | 1.000 | 1.000 |
| 1 min β 150 | 1.5 min β 300 | 0.074 | 1.000 | 0.043 |
| 1 min β 150 | 1.5 min β 400 | 0.415 | 1.000 | <0.001 |
| 1 min β 150 | 1.5 min β 500 | 1.000 | <0.001 | <0.001 |
| 1 min β 150 | 1.5 min β 550 | 1.000 | <0.001 | <0.001 |
| 1 min β 150 | 2 min β 150 | 0.814 | 1.000 | 1.000 |
| 1 min β 150 | 2 min β 200 | 0.043 | 1.000 | 0.584 |
| 1 min β 150 | 2 min β 300 | 0.005 | 1.000 | 0.001 |
| 1 min β 150 | 2 min β 400 | 0.029 | 1.000 | <0.001 |
| 1 min β 150 | 2 min β 500 | 0.466 | <0.001 | <0.001 |
| 1 min β 150 | 2 min β 550 | 1.000 | <0.001 | <0.001 |
| 1 min β 200 | 1 min β 500 | 1.000 | 1.000 | 0.025 |
| 1 min β 200 | 1 min β 550 | 1.000 | <0.001 | 0.001 |
| 1 min β 200 | 1.5 min β 300 | 1.000 | 1.000 | 0.415 |
| 1 min β 200 | 1.5 min β 400 | 1.000 | 1.000 | 0.002 |
| 1 min β 200 | 1.5 min β 500 | 1.000 | 0.001 | <0.001 |
| 1 min β 200 | 1.5 min β 550 | 1.000 | <0.001 | <0.001 |
| 1 min β 200 | 2 min β 300 | 0.370 | 1.000 | 0.022 |
| 1 min β 200 | 2 min β 400 | 1.000 | 1.000 | <0.001 |
| 1 min β 200 | 2 min β 500 | 1.000 | <0.001 | <0.001 |
| 1 min β 200 | 2 min β 550 | 1.000 | <0.001 | <0.001 |
| 1 min β 300 | 1 min β 550 | 1.000 | 0.079 | 0.292 |
| 1 min β 300 | 1.5 min β 400 | 1.000 | 1.000 | 0.584 |
| 1 min β 300 | 1.5 min β 500 | 1.000 | 0.466 | 0.004 |
| 1 min β 300 | 1.5 min β 550 | 1.000 | 0.027 | <0.001 |
| 1 min β 300 | 2 min β 400 | 1.000 | 1.000 | 0.003 |
| 1 min β 300 | 2 min β 500 | 1.000 | 0.050 | <0.001 |
| 1 min β 300 | 2 min β 550 | 1.000 | 0.002 | <0.001 |
| 1 min β 400 | 2 min β 550 | 1.000 | 0.349 | 1.000 |
| 1 min β 500 | 1.5 min β 150 | 1.000 | 1.000 | 0.050 |
| 1 min β 500 | 1.5 min β 300 | 0.522 | 1.000 | 1.000 |
| 1 min β 500 | 2 min β 200 | 0.329 | 0.244 | 1.000 |
| 1 min β 500 | 2 min β 300 | 0.050 | 1.000 | 1.000 |
| 1 min β 550 | 1.5 min β 150 | 1.000 | <0.001 | <0.001 |
| 1 min β 550 | 1.5 min β 200 | 0.259 | <0.001 | 0.084 |
| 1 min β 550 | 1.5 min β 300 | 0.050 | 0.124 | 1.000 |
| 1 min β 550 | 1.5 min β 400 | 0.292 | 1.000 | 1.000 |
| 1 min β 550 | 2 min β 150 | 0.584 | <0.001 | 0.140 |
| 1 min β 550 | 2 min β 200 | 0.029 | 0.010 | 1.000 |
| 1 min β 550 | 2 min β 300 | 0.003 | 1.000 | 1.000 |
| 1 min β 550 | 2 min β 400 | 0.019 | 1.000 | 1.000 |
| 1 min β 550 | 2 min β 500 | 0.329 | 1.000 | 1.000 |
| 1.5 min β 150 | 1.5 min β 300 | 1.000 | 1.000 | 0.730 |
| 1.5 min β 150 | 1.5 min β 400 | 1.000 | 1.000 | 0.005 |
| 1.5 min β 150 | 1.5 min β 500 | 1.000 | <0.001 | <0.001 |
| 1.5 min β 150 | 1.5 min β 550 | 1.000 | <0.001 | <0.001 |
| 1.5 min β 150 | 2 min β 300 | 1.000 | 1.000 | 0.043 |
| 1.5 min β 150 | 2 min β 400 | 1.000 | 1.000 | <0.001 |
| 1.5 min β 150 | 2 min β 500 | 1.000 | <0.001 | <0.001 |
| 1.5 min β 150 | 2 min β 550 | 1.000 | <0.001 | <0.001 |
| 1.5 min β 200 | 1.5 min β 400 | 1.000 | 1.000 | 0.180 |
| 1.5 min β 200 | 1.5 min β 500 | 1.000 | 0.002 | 0.001 |
| 1.5 min β 200 | 1.5 min β 550 | 1.000 | <0.001 | <0.001 |
| 1.5 min β 200 | 2 min β 400 | 1.000 | 1.000 | 0.001 |
| 1.5 min β 200 | 2 min β 500 | 1.000 | <0.001 | <0.001 |
| 1.5 min β 200 | 2 min β 550 | 1.000 | <0.001 | <0.001 |
| 1.5 min β 300 | 1.5 min β 500 | 1.000 | 0.690 | 1.000 |
| 1.5 min β 300 | 1.5 min β 550 | 1.000 | 0.043 | 1.000 |
| 1.5 min β 300 | 2 min β 500 | 1.000 | 0.079 | 1.000 |
| 1.5 min β 300 | 2 min β 550 | 1.000 | 0.003 | 1.000 |
| 1.5 min β 400 | 2 min β 550 | 1.000 | 0.618 | 1.000 |
| 1.5 min β 500 | 2 min β 150 | 1.000 | 0.001 | 0.001 |
| 1.5 min β 500 | 2 min β 200 | 1.000 | 0.079 | 0.124 |
| 1.5 min β 550 | 2 min β 150 | 1.000 | <0.001 | <0.001 |
| 1.5 min β 550 | 2 min β 200 | 1.000 | 0.003 | 1.000 |
| 1.5 min β 550 | 2 min β 300 | 1.000 | 0.653 | 1.000 |
| 2 min β 150 | 2 min β 400 | 1.000 | 1.000 | 0.001 |
| 2 min β 150 | 2 min β 500 | 1.000 | <0.001 | <0.001 |
| 2 min β 150 | 2 min β 550 | 1.000 | <0.001 | <0.001 |
| 2 min β 200 | 2 min β 500 | 1.000 | 0.006 | 1.000 |
| 2 min β 200 | 2 min β 550 | 1.000 | <0.001 | 1.000 |
| 2 min β 300 | 2 min β 550 | 1.000 | 0.79 | 1.000 |
